# Prolonged California aridity linked to climate warming and Pacific sea surface temperature

**DOI:** 10.1038/srep33325

**Published:** 2016-09-15

**Authors:** Glen M. MacDonald, Katrina A. Moser, Amy M. Bloom, Aaron P. Potito, David F. Porinchu, James R. Holmquist, Julia Hughes, Konstantine V. Kremenetski

**Affiliations:** 1 Department of Geography, University of California, Los Angeles, 1255 Bunche Hall, Los Angeles, CA 90095-1524, USA; 2 Department of Geography and Centre for Environment and Sustainability, University of Western Ontario, 1151 Richmond St., North, London, Ontario, N6A 5C2, Canada; 3 Department of Geography-Geology, Illinois State University, Campus Box 4400, Normal, IL 61790-4400, USA; 4 School of Geography and Archaeology, National University of Ireland, Galway, Galway, Ireland; 5 Department of Geography, University of Georgia, 210 Field Street, Athens, GA 30602-001, USA; 6 Smithsonian Environmental Research Center, Edgewater, MD, 21037-0028, USA; 7 Westminster School, Geography, London, SW1P 3PF, UK

## Abstract

California has experienced a dry 21^st^ century capped by severe drought from 2012 through 2015 prompting questions about hydroclimatic sensitivity to anthropogenic climate change and implications for the future. We address these questions using a Holocene lake sediment record of hydrologic change from the Sierra Nevada Mountains coupled with marine sediment records from the Pacific. These data provide evidence of a persistent relationship between past climate warming, Pacific sea surface temperature (SST) shifts and centennial to millennial episodes of California aridity. The link is most evident during the thermal-maximum of the mid-Holocene (~8 to 3 ka; ka = 1,000 calendar years before present) and during the Medieval Climate Anomaly (MCA) (~1 ka to 0.7 ka). In both cases, climate warming corresponded with cooling of the eastern tropical Pacific despite differences in the factors producing increased radiative forcing. The magnitude of prolonged eastern Pacific cooling was modest, similar to observed La Niña excursions of 1^o^ to 2 °C. Given differences with current radiative forcing it remains uncertain if the Pacific will react in a similar manner in the 21st century, but should it follow apparent past behavior more intense and prolonged aridity in California would result.

In the 21^st^ century California has experienced arid conditions and the most intense drought on record; 10 of the last 14 years have been drier than normal, and the last three have been the hottest and driest since 1895^1^. At the same time, tropical easterlies over the Pacific strengthened and eastern Pacific SST’s declined relative to the western Pacific potentially contributing to this aridity^2^. Although the high temperatures associated with the current drought reflect increasing greenhouse gases^3^, the low precipitation is within the bounds of past natural variability^4^,^5^. Instrumental meteorological data for the past century and tree-ring records of the past millennium indicate that prolonged droughts in California are typically associated with reduced precipitation correlated to a ridge of atmospheric high pressure over the North Pacific with warm surface temperatures enhancing evapotranspiration^4^,^5^,^6^. Due largely to the Pacific Ocean teleconnection it remains uncertain how California hydroclimatology will respond to future greenhouse gas forcing. Based on current observational data and models, warming or cooling of the eastern Pacific remains possible and could either mitigate or exacerbate aridity in California^2^,^7^. Research on the current drought is also ambiguous about the effects of anthropogenic warming on Pacific SST’s and storm tracks^1^.

Instrumental records are limited in length and ability to capture the full range of natural hydroclimatic variability. Highly resolved (decadal or less) lake sediment studies from sensitive sites can provide multi-millennial records of temperature, droughts and oceanic linkages^8^. Here we reconstruct a hydroclimatic history that spans the past ~10 ka based upon multi-proxy analyses of a sediment record from hydroclimatologically sensitive Kirman Lake in central-eastern California (Fig. 1). The hydroclimatology of the region and relation to Pacific Ocean forcing is representative of California in general and the Sierra Nevada where 60% of California’s managed water originates. A significant correlation exists between the NINO 3.4 SST anomalies and the Pacific Decadal Oscillation (PDO) index and annual precipitation at the Kirman Lake region and these local values are representative of the gridded correlations between these measures of Pacific variability and precipitation over the Sierra Nevada and adjacent portions of California (See Supplementary Information and Supplementary Fig. S1). Comparison with other proxy climate data and paleoceanographic reconstructions from the tropical and extra-tropical Pacific^9^,^10^,^11^ demonstrates a linkage in the past between positive radiative forcing events, climate warming, cooling of the eastern tropical Pacific and increased aridity in central-eastern California. In contrast, coincident with cooling during the 4.2 ka event^12^ and the general neoglacial cooling following ~3 ka the opposite reaction occurred with a relative warming of the eastern Pacific and increased moisture in California.

## Results

### Kirman Lake: A Record of California Aridity

The most notable feature of the 3.1 m sediment stratigraphy from Kirman Lake is a multi-millennial arid period from ~8 to 3 ka. During this time diatom-inferred salinity and depth indicate that Kirman Lake was shallow and characterized by increased salinity (Fig. 2). High abundances of the diatom *Gomphonema angustum* (see Supplementary Fig. S4) support an interpretation of expanded marsh. *Gomphonema angustatum* is observed growing on *Scirpus acutus* var. *occidentalis* (common tule), the dominant plant in the marsh of Kirman Lake. Replacement of open water by marsh is also consistent with the elemental and isotopic records. The increased sediment organics, δ^13^C values (−275‰ to −32‰), C:N values (~15 to 20) and depleted δ^15^N during this period are consistent with algae combined with increasing vascular marsh plants as the primary carbon source to the sediments (Fig. 2)^13^,^14^. Mollusks disappear during this interval, which is expected because of loss of non-vegetated benthic habitat and decreased preservation in more organic, lower pH sediments (Fig. 2). A surface sample transect across Kirman Lake shows high organic content and low mollusk abundance in shallow marsh fringes of the lake relative to the deeper, open water area (Supplementary Information and Supplementary Fig. S2). The fossil pollen and charcoal records also indicate enhanced aridity between 8 to 3 ka (Fig. 2). The depression of *Pinus:Artemisia* suggests a decrease of mesic pine woodlands in favor of more xerophytic sagebrush. The high background values and peaks in charcoal suggest fires of greater spatial extent and/or intensity relative to prior and subsequent fire regimes. In addition, all proxies suggest decreased amplitude in hydroclimatic variability during the 8 to 3 ka period (Fig. 2). However, a notable and discrete excursion to greater lake depths and lower salinity occurs ~4.2 ka (Figs 2 and 3).

**Figure 1:**
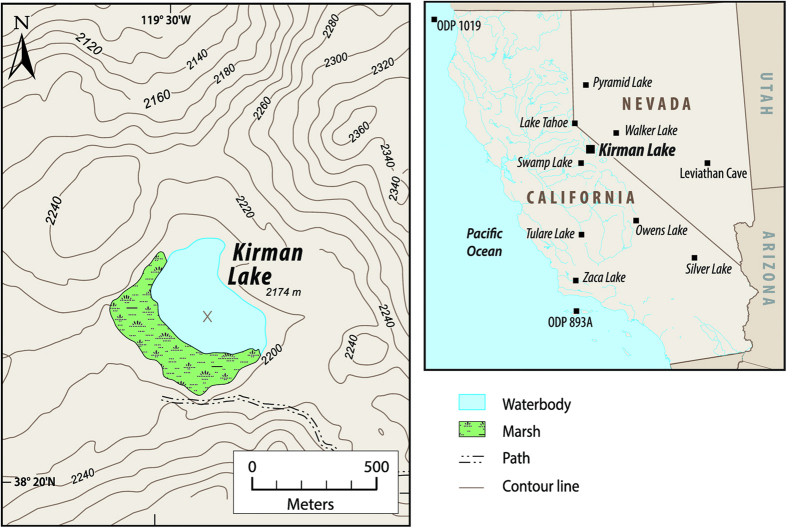
Location and topographic setting of Kirmin Lake, California and location of regionally adjacent sites mentioned in text. Map created by GMM and UCLA Geography from United States Geological Survey (USGS) topographic data http://nationalmap.gov/elevation.html using Adobe CS6 (Mac) http://www.adobe.com/products/illustrator.html; Avenue Mapublisher 9.6 (Mac) http://www.avenza.com/mapublisher and Natural Scene Designer 6.0 (Mac) https://www.naturalgfx.com.

Supporting evidence for aridity in the Sierra Nevada and adjacent areas between ~8 to 3 ka can be found at a number of other sites (Fig. 1)^15^. Lowstands occur at Pyramid Lake, Nevada and Owens Lake, California beginning ~8 ka with a shift towards moister conditions between ~5 and 3 ka. Walker Lake, located between Kirman Lake and Pyramid Lake, shows evidence of desiccation between at least 5.5 and 2.7 ka. Drier conditions between 8 and 3 ka have been reconstructed from drowned forest at Lake Tahoe^15^. Fossil pollen and charcoal data from the Sierra Nevada indicate drier, more open forests in the early through middle Holocene and moister, more closed forest with decreased fire frequency following 3 ka^16^. Increased sand and mud cracks in sediment records from Silver Lake, California between ~7.5 and 4 ka and lowstands at Tulare Lake, California between 7.8 to 5.5 ka also indicate a prolonged period of mid-Holocene aridity^17^,^18^. Warmer temperatures occurred between 8 to 3 ka and could have played a role in depressing effective moisture. Chironomid-inferred air temperatures for the Sierra Nevada and Great Basin indicate summer air temperatures between 8-3 ka were 1 to 2 °C warmer than present^19^, which is consistent with elevated treeline observed in the Sierra Nevada and nearby White Mountains at this time^20^. Warmer mid-Holocene temperatures are also supported by higher δ^18^O in a speleothem record from nearby Levithan Cave, Nevada^21^.

**Figure 2:**
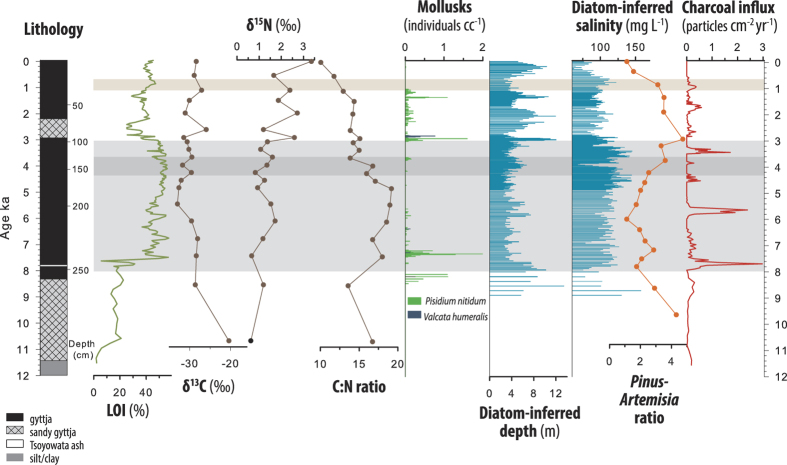
General sediment stratigraphy, organic content as represented by loss-on-ignition at 550 °C (LOI), δ^13^C, δ^15^N and C:N stratigraphies, sediment mollusk concentrations, diatom-inferred depth and salinity, *Pinus:Artemisia*, and charcoal accumulation rates. Dry mid-Holocene indicated with light grey shading, 4.2 ka wet interval indicated by dark grey shading. Medieval Climate Anomaly (MCA) dry interval is indicated with tan shading.

**Figure 3:**
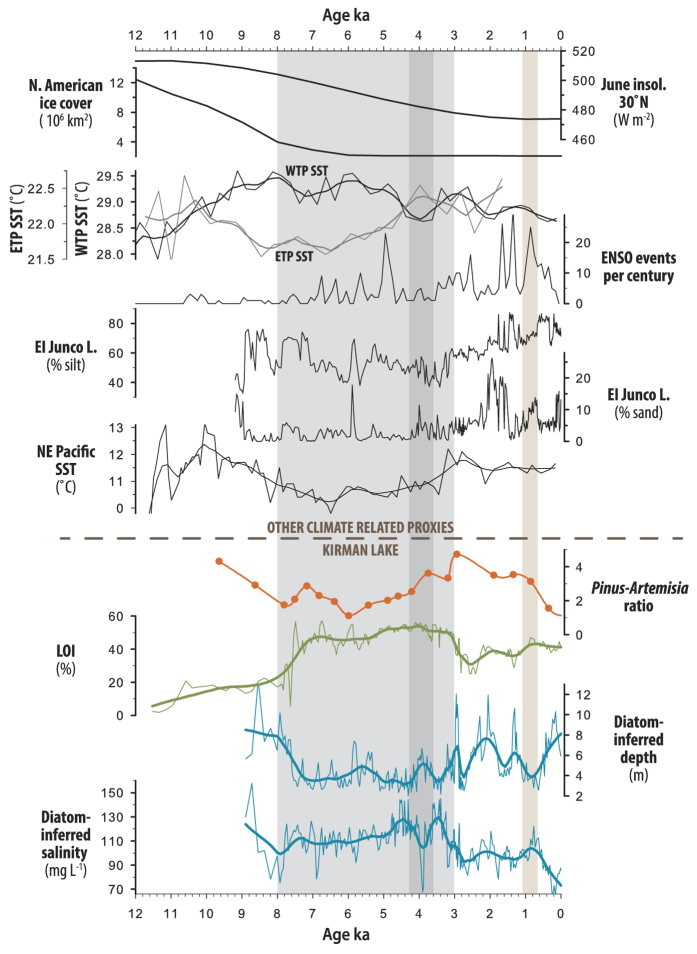
North American Ice cover^39^, orbitally induced solar forcing^40^, eastern tropical Pacific (ETP - green) and western tropical Pacific (WTP - orange) sea surface temperatures (SST’s)^9^,^10^ from foraminifera, ENSO events per century inferred from South American lake sediments^41^, record of eastern tropical Pacific precipitation inferred from Galapagos Island lake sediment silt content and of ENSO frequency inferred from Galapagos Island lake sediment sand content^25^, alkenone based reconstruction of eastern North Pacific SST’s from ODP1019^11^ (Fig. 1), Kirman Lake Pinus-Artemisia pollen ratio, LOI and diatom-inferred depth and salinity. Dry mid-Holocene period indicated with light grey shading, 4.2 ka wet interval indicated by dark grey shading. Medieval Climate Anomaly (MCA) dry interval is indicated with tan shading. Heavier overlay lines represent LOESS (span 0.10) smoothed series.

Although aridity returns after the 4.2 ka event, in the period between 4 and 3 ka there is a gradual shift to moister conditions at Kirman Lake as evidenced by increasing lake levels and declining salinity (Figs 2 and 3). After ~3 ka conditions are generally wetter and also more variable than during the mid-Holocene. This interpretation is supported by decreased sediment organics and increased mollusk content, which would result from a reduction in marsh conditions as lake levels rose and a decrease in C:N values indicating an increase in algae relative to emergent vascular plants contributing to organic sediments^13^. Increasing moisture in the Kirman Lake region is also suggested by a decrease in *Artemesia* pollen relative to *Pinus* and decreased charcoal accumulation (Fig. 2). A variety of paleo-hydroclimatic proxies similarly show that between ~4 ka to 3 ka there was a shift to wetter and cooler conditions at sites across California and in the adjacent Great Basin (Fig. 1)^8^,^17^,^18^,^21^,^22^.

**Figure 4:**
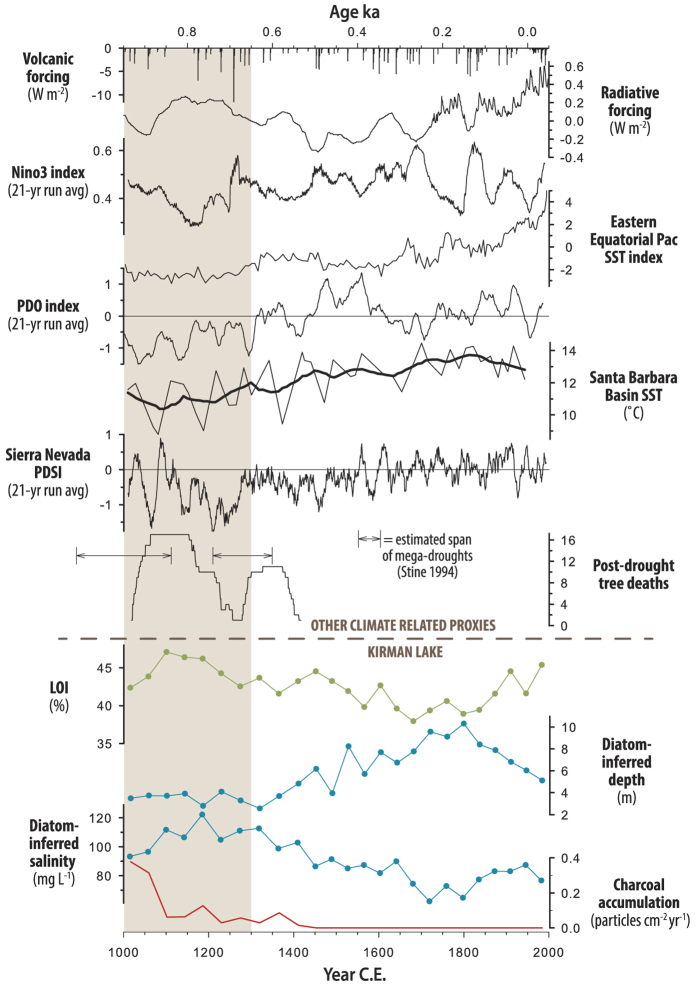
Volcanic activity and solar and greenhouse gas and particulate radiative forcing^42^, modeled response of Nino3^43^, eastern equatorial Pacific sea surface temperature (SST)^44^, Pacific Decadal Oscillation (PDO) index^45^, Santa Barbara Channel SST from ODP893A^46^ (Fig. 1), Sierra Nevada Palmer Drought Severity Index (PDSI)^47^, estimate of eastern California mega-droughts based on submerged tree-stumps^48^, Kirman Lake loss-on-ignition (LOI), diatom-inferred depth and salinity and charcoal accumulation. Medieval Climate Anomaly (MCA) dry interval is indicated with tan shading. Heavier overlay lines represent LOESS (span 0.10) smoothed series.

Our lake level and salinity data show that the generally wetter period following ~3 ka was punctuated by markedly drier periods that persisted for hundreds of years. Such events were also reported in a high temporal resolution record from Zaca Lake in southern California^8^. One of these distinct dry periods is coincident with the Medieval Climate Anomaly (MCA ~1 ka to 0.7 ka)^23^, which is observed in many California paleoclimate records^24^. Kirman Lake experienced high salinity, decreased depth and increased organic sedimentation during the MCA (Figs 2, 3 and 4) and a reversal of these conditions during the subsequent Little Ice Age (LIA ~0.7 ka to 0.16 ka) (Fig. 4). Numerous lines of evidence demonstrate that generally increased aridity and higher temperatures during the MCA extended over California, the Southwest and the Colorado River basin during the MCA (Fig. 4)^6^,^24^.

### Connections between California Aridity and the Pacific Ocean

Evidence of the Pacific Ocean as a forcing agent contributing to prolonged aridity between ~8 ka to 3 ka, the excursion to wetter conditions during the 4.2 ka event and greater variability during the late Holocene can be identified in Pacific Ocean SST variations (Fig. 3)^25^,^26^. The period of 8 to 3 ka is typified by depression of tropical Pacific SST’s in the east and elevated SST’s in the west. This is analogous to a multi-millennial La Niña conducive to enhanced aridity in California. Pacific SST’s along the California coastline were also depressed between 8 to 3 ka and provide evidence of an extratropical extension of eastern Pacific cooling (Fig. 3). The extratropical extension of eastern Pacific cooling is similar in nature to a prolonged negative state of the Pacific Decadal Oscillation (PDO) and would have been additionally conducive to enhanced aridity in California^27^.

The lake deepening and freshening during the 4.2 ka event is contemporaneous with evidence of a discrete oceanic event typified by warming in the eastern Pacific and corresponding cooling in the western Pacific (Fig. 3). Hydrological changes reflecting increased aridity during the 4.2 ka event have been widely observed in the Middle East, India and China and have been correlated with cooling of the western Pacific and warming of the eastern Pacific and associated with boreal cooling and moister conditions in western North America^26^,^28^,^29^. California and India generally display an opposite response to El Niño-Southern Oscillation (ENSO) variations, and increased moisture in California at the same time that aridity increases in India provides strong evidence for the importance of the Pacific Ocean in the hydroclimatological changes associated with the 4.2 ka event in the two regions^26^. Similar to the 4.2 ka event, the last 3 ka have been generally wetter than the mid-Holocene, to which warmer eastern Pacific SST’s would have contributed (Fig. 3)^22^.

The higher amplitude hydroclimatic excursions of the past 3 ka at Kirman Lake correspond to similar changes reported from Zaca Lake and increased variability in ENSO (Fig. 3) which is also manifest in higher amplitude hydroclimatic variability at the opposite latitudinal dipole in India^8^,^26^. High-resolution analysis of the past 1,000 years and the impacts of the MCA at Kirman Lake and from other California and Pacific Ocean paleorecords provide further evidence of the sensitivity of eastern Pacific SST’s and California hydroclimatology to warming (Fig. 4). The MCA drying widely experienced in California is contemporaneous with apparent cooling in the eastern equatorial Pacific and eastern North Pacific coastal waters and a negative state of the PDO (Fig. 4)^24^,^30^,^31^.

## Discussion

The protracted dry period in California and cooling of the eastern Pacific between 8 to 3 ka and during the MCA, and brief wet period and warming of the eastern Pacific at 4.2 ka underscore the potential sensitivity of California and the Pacific to prolonged excursions in radiative forcing. The inferred long-term warming of the eastern tropical Pacific and cooling in the western tropical Pacific were on the order of 1–2 °C over the 8 to 3 ka period and the MCA, whereas the positive excursion in eastern Pacific SST’s during the 4.2 ka event linked to moister conditions in California was about half that magnitude (Fig. 3). For comparison, in the Nino3.4 area (5° S–5° N, 120° W–170° W) the annual SST anomalies, robustly filtered to remove other annual effects, for the 14 strongest La Niña events from CE 1856**–**2009 ranged between −0.91 and −1.48 °C^32^. Thus, it is the prolonged nature of the SST anomalies, rather than extraordinary magnitude that is exceptional in these earlier Holocene events. If such prolonged, but not especially high-magnitude, events are associated with exceptional aridity or increased moisture in the past, they would likely be capable of contributing to prolonged aridity in the future.

The prolonged cooling of the eastern Pacific and limited ENSO-linked variability in the early to mid-Holocene were likely ultimately driven by orbitally-induced insolation changes^33^. The 8 to 3 ka interval coincides with relatively high values in summer insolation over the Northern Hemisphere. Although maximum summer insolation in the Northern Hemisphere occurred earlier, it was not until ~8 to 7 ka that the final decay of the Laurentide Ice Sheet produced current boundary conditions and allowed a greater proportion of insolation to contribute to surface heating (Fig. 3). However, modeling shows that increased summer insolation alone is unlikely to have caused the increase in aridity during this time and suggests atmospheric-oceanic linkages are important^34^. Lack of high magnitude ENSO-style variability and associated eastern Pacific warming may be linked to the high boreal summer insolation and low equatorial seasonality in the early to mid-Holocene^35^. Through a number of feedbacks this, coupled possibly with a northward extension of the Inter-Tropical Convergence Zone and an enhanced Indian Ocean-western Pacific warm pool, increased zonal SST and pressure gradients and drove the Pacific towards limited ENSO-style variability^35^. Cooling during the early to mid-Holocene extended to the extratropical Pacific off the California coast (Fig. 3) and potentially generated persistent negative PDO-like conditions, exacerbating California aridity. There is extensive evidence that between 4 and 3 ka these oceanic conditions terminated as boreal summer insolation declined. At this time ENSO-teleconnected regions around the Pacific began to experience ENSO-related variability similar to today^25^.

Changes in Pacific SST’s and California hydroclimatology during the MCA can be linked to relatively modest radiative forcing related to increased solar output and decreased volcanic activity (Fig. 4). Increased radiative forcing at this time produced warming that increased longitudinal gradients in the equatorial Pacific and appears to have led to development of persistent La Niña-like conditions and a negative state of the PDO. Warmer temperatures coupled with cooling of the eastern tropical and equatorial Pacific would have resulted in the persistent aridity observed at Kirman Lake (Fig. 4) and across California^8^.

The Kirman Lake record shows that central eastern California, and by extension in California and adjacent areas in the Great Basin, have exhibited a positive relationship between large-scale climate warming, cooling of the eastern tropical and extra-tropical Pacific and prolonged drought through the Holocene^8^,^17^,^18^,^21^,^22^. IPCC estimates for global radiative forcing under best to worst-case scenarios for 2100 range from 2.5 to 8.5 Wm^−2^ 36. Although lower in seasonally and regionally specific magnitudes than the orbitally induced seasonal/latitudinal radiative changes of the middle Holocene (Fig. 3), these figures can be compared to global net positive radiative forcing during the MCA which was associated with surface warming, prolonged cooling of the eastern Pacific and extended aridity in California (Fig. 4). Taken together, paleoclimatological and paleoceanographic records indicate a system sensitive to radiative forcing which can generate centennial or longer changes in the hydroclimatology of California. As greenhouse gas forcing differs from the earlier climate forcing in terms of mechanisms, wave-lengths affected and seasonal and latitudinal properties, so to the ultimate response of the Pacific may differ. Although climate modeling experiments suggest that current forcing by increased greenhouse gasses may produce an opposite oceanic response in the future, much uncertainty remains in such estimates^7^,^37^. However, as both model experiments and the paleorecords presented here demonstrate the potential sensitivity of the Pacific Ocean and California climate to radiative forcing, the response of the Pacific and the ENSO system over the 21^st^ century remains in need of greater understanding in order to fully anticipate the effects of increasing greenhouse gasses on California’s hydroclimatology.

## Methods

### Study Site

A multi-proxy, paleolimnological approach was used at Kirman Lake (38°20′24″ N; 119°29′59″ W; 2,174 m asl). The lake has a surface area of ~12 ha and a maximum depth of ~4 m with a gently sloping bottom bathymetry. A marsh dominated by *Scirpus acutus* var. *occidentalis* is present at the western and southern edges of the lake (Fig. 1). Kirman Lake is an ideal site for determining past aridity because it is a closed basin lake in a climatically sensitive central region representative of California hydrology. Lake levels and salinity of closed-basin lakes are largely controlled by changes in effective moisture; therefore, proxies of lake level and salinity, including diatoms and geochemical measures, can be used to infer periods of past aridity. Because vegetation and fire occurrence are strongly influenced by climate, analysis of pollen and charcoal in the lake sediments provides additional evidence of periods of dry conditions.

Climate at the lake, as represented by NOAA Station CA041072 24 km to the east, is semi-arid with a mean annual precipitation of 23 cm concentrated in November through April and average January and July temperatures of −4.3 and 16.0 °C. Precipitation in the region is sensitive to Pacific SST variability typical of ENSO and the lower frequency PDO, characterized by a positive correlations between NINO 3.4 SST’s, the annual PDO index and annual precipitation in the region. Positive PDO values reflect warmer conditions in the eastern extratropical North Pacific and are associated with and influenced by positive phases of ENSO (See Supplementary Information and Supplementary Fig. S1).

The catchment vegetation is characteristic of the ecotone between the lower montane conifer forests of the Sierra Nevada, which includes *Pinus jeffreyi* (Jeffrey pine) and *Pinus monophylla* (pinyon pine), and Great Basin sagebrush typified by common occurrence of *Artemisia tridentate* (big sagebrush). Slopes to the southwest contain open groves of pine while the areas immediately around the lake and lower in elevation to the east are dominated by graminoids, herbs and low shrubs.

### Sediment Core Acquisition and Analysis

A sediment core was retrieved from Kirman Lake on July 18, 2000, using a modified Livingstone piston corer from a fixed position raft near the center of the lake (Fig. 1, Supplementary Information). Sediment ages were determined from an age-depth model created based on seven AMS ^14^C dates and a tephra date using a flexible Bayesian approach (see Supplementary Information and Supplementary Table 1 and Fig. S3). Diatom-inference models (see Supplementary Information) were used to determine past lake depth and salinity. These models are based on a 57-lake calibration set that spans the eastern side of the Sierra Nevada^38^ (see Supplementary Information). Variations in mollusk shell abundance, percentage organics and sediment geochemistry (δ^13^C, δ^15^N and C:N ratios) were used to track changes in the coverage of marsh versus open water. These proxies were measured using standard preparation and analytical techniques that are described fully in the Supplementary Information section. Pollen and charcoal analyses were used to determine changes in vegetation and fire frequency. Details of these analyses are also provided in the Supplementary Information section. Our long-term record of California aridity was compared to other records from California and the Great Basin, as well as records of Pacific sea surface temperatures, records of ENSO and PDO and reconstructed orbital and radiative forcings.

## Additional Information

**How to cite this article**: MacDonald, G. M. *et al*. Prolonged California aridity linked to climate warming and Pacific sea surface temperature. *Sci. Rep.* **6**, 33325; doi: 10.1038/srep33325 (2016).

## Supplementary Material

Supplementary Information
