# Supplementary material for: Prolonged California aridity linked to climate warming and Pacific sea surface temperature
Source: Sci Rep. 2016 Sep 15;6:33325. doi: 10.1038/srep33325 (PMC5024308; doi:10.1038/srep33325)
Supplement: Supplementary Information [file srep33325-s1.pdf]

Supplementary Information

Prolonged California aridity linked to climate warming and Pacific sea surface temperature

Glen M. MacDonald[1*], Katrina A. Moser[2], Amy M. Bloom[3], Aaron Potito[4], David F. Porinchu[5], James R. Holmquist[6], Julia Hughes[7] and Konstantine V. Kremenetski[1]

[1]Department of Geography, Los Angeles, 1255 Bunche Hall, Los Angeles, CA 90095-1524, USA (macdonal@geog.ucla.edu); [2]Department of Geography and Centre for Environment and Sustainability, University of Western Ontario, 1151 Richmond St., North, London, Ontario, N6A 5C2, Canada; [3]Department of Geography-Geology, Illinois State University, Campus Box 4400, Normal, IL 61790-4400, USA; [4]School of Geography and Archaeology, National University of Ireland, Galway, Galway, Ireland; [5]Department of Geography, University of Georgia, 210 Field Street, Athens, GA 30602-001, USA; Smithsonian Environmental Research Center, Edgewater, MD, 21037-0028, USA; Westminster School, Geography, London, SW1P 3PF, UK.

[*]Corresponding Author

Supplementary Methods and Details of Results

Site Climatic Sensitivity and Representativeness

The Kirman Lake lies in California Climate Division 3 which encompasses the eastern flank of the Sierra Nevada and adjacent Great Basin. The region is sensitive to Pacific SST variability typical of ENSO and the lower frequency Pacific Decadal Oscillation PDO[1]. This is characterized by significant positive correlations between Niño 3.4 SST’s, the PDO index and winter precipitation (November-April) for California Climate Division 3 (1901-2015 NINO3.4 $r= 0.230$ $p \leq 0.05$, PDO $r= 0.237$ $p \leq 0.05$. The positive relationships between eastern Pacific tropical and extra-tropical SST’s observed in the Kirman Lake region are similar to those observed in gridded data for the Sierra Nevada in general and much of the rest of California (Supplementary Fig. S1). Positive PDO values reflect warmer conditions in the eastern extratropical North Pacific and are associated with and influenced by positive phases of ENSO[2]. A significant positive correlation exists between November-April SST’s in NINO3.4 and the PDO index (1901-2015 $r= 0.48$, $p\leq 0.05$).

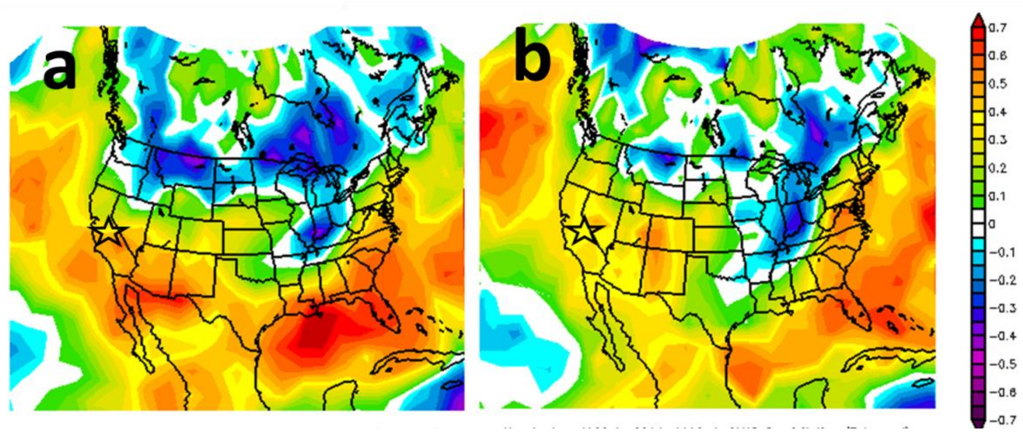

Supplementary Figure S1. The location of Kirman Lake (☆) and the correlations between November-April precipitation and a. November-April Niño 3.4 SST’s and b. November-

April PDO index – 1980-2015 (Analysis by GMM, UCLA Geography, using data and online mapping application software provided by NOAA Earth Systems Research Laboratory (ESRL) http://www.esrl.noaa.gov/psd/data/correlation/ . Data/image provided by the NOAA/OAR/ESRL PSD, Boulder, Colorado, USA, from their Web site at http://www.esrl.noaa.gov/psd/ .

Sediment Coring and Sediment Core

Lake sediment was recovered from the centre of the lake at a depth of 4 m using a modified Livingstone piston corer from a raft (Supplementary Fig. S2)[3]. The uppermost sediment was recovered with a 1 m long plastic tube attached to the corer to ensure collection of an undisturbed sediment-water interface. Promptly after recovery, the plastic tube sediments were extruded on shore in 1.0 cm intervals and placed into individual plastic bags. Underlying stiffer sediment was collected in aluminum alloy core barrels, extruded at 1 m intervals from the core barrel intact, wrapped in plastic film and aluminum foil while on the coring platform, and placed in wooden core boxes. All samples were kept dark and cool until shipped to the laboratory where they were refrigerated at 4°C. A sediment record 310.0 cm long was retrieved from Kirman Lake. The lowermost part of the core (~269.0-310.0 cm) is comprised of silty clay, while the upper 269.0 cm of the core is organic lake sediment. A layer of silty-sandy organic-rich sediment is present from ~252 -269.0 cm and a layer of sandy organic lake sediment is present from ~63.0-90.0 cm.

Surface Sediment Sampling Transect

In order to determine the modern distribution of sediment organic content and habitat preferences for mollusc taxa to enhance our paleohydrologcial interpretation (Supplementary Fig. S2), surface sediments in Kirman Lake were collected using an Ekman dredge. Loss-on-ignition (LOI) analysis indicates that highest sediment organic content is found in the shallow (<1 m) *Scirpus* marsh zone. Modern freshwater mollusc assemblages were strongly associated with submerged aquatics, particularly *Chara*. There were moderate amounts of *Pisidium nitidum* and *Valvata humeralis* along the sandy shallow lake edges and in the relatively shallow (1-3 m) peripheral open water zone just beyond the *Scirpus* marsh (Supplementary Fig. S2). Both species were rare to absent in the intervening *Scirpus* vegetation zone, likely due to high acidity in the vegetation mat[4] and *P. nitidum*'s general rarity among dense stands of emergent plants[5]. Both mollusc species reach peak abundances at approximately 1-2 m depth where benthic *Chara* concentrations are at their highest, and both are rarer in deeper (4 m) non-vegetated sediments in the deepest portion of the modern lake beyond the photic zone. The sporadic presence of *Chara* oospores throughout the core indicates its presence in the lake through much of the Holocene. The high organic content during the period 8 to 3 ka is consistent with shallowing and expansion of marsh cover as is the general absence of molluscs. The mollusc peaks in the sedimentary record tend to coincide with reconstructed deeper water conditions with a reduced marsh cover.

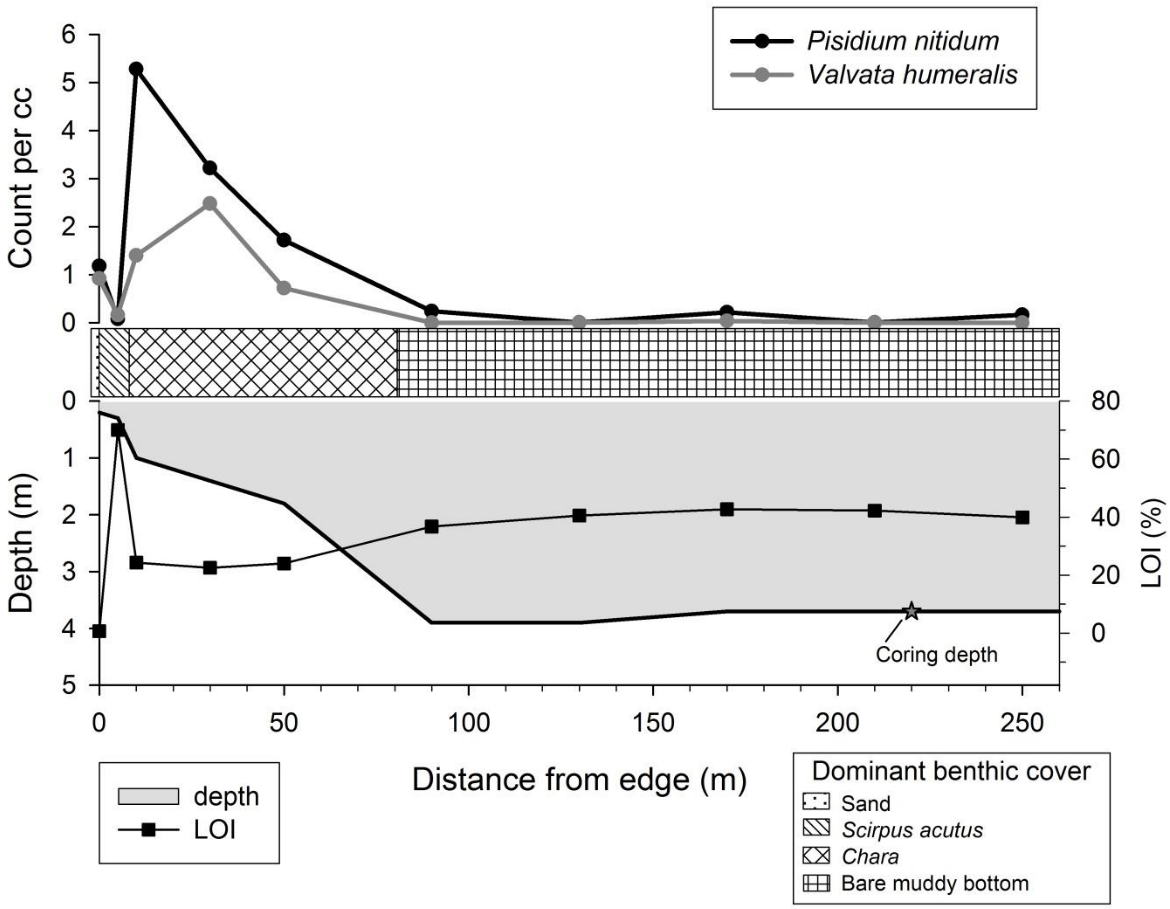

Supplementary Figure S2. Mollusc abundances, dominant benthic cover and loss-on-ignition (LOI) results from a surface sample transect using an Ekman dredge. Coring depth is indicated by a star.

Chronologic Control

Seven AMS ^{14}C dates were obtained from bulk organics at UC Irvine, Beta Analytic, and the National Science Research Laboratory (Supplementary Table S1). The Tsoyawata Tephra occurred at 247.0 – 248.5 cm, and was identified by F. Foit, Jr. at the Microbeam Facility at the Washington State GeoAnalytical Laboratory and the ^{14}C date for the Tsoyawata tephra was

previously determined[6]. The sediment surface and year of collection [-51.6 cal yr BP] was used as a data point, with an assumption of ± 5 years uncertainty. As no terrestrial macrofossils were available bulk organics were used for dating. Any error from old carbon is expected to be minimal because: 1) the underlying geology is comprised of Mesozoic granites and Tertiary volcanics with little to no carbonate; 2) the catchment to lake area is relatively small (~10) and there are no inflows to the lake ensuring a relatively small contribution of catchment materials that could potentially contain old carbonate from the surficial alluvium, and 3) other than the bottom date, which is older than anticipated, the dates are consistent with the volcanic ash (Supplementary Table S1, Supplementary Fig. S3).

Supplementary Table S1. Radiocarbon date and Tsoyawata Tephra stratigraphy at Kirman Lake.

Lab I.D.	Depth (cm)	Material	14C Age	95% Limits (cal yr BP)	Weighted Mean Age (cal yr BP)
UCIAMS115838	26.0-27.0	Bulk Sediment	1130±20	961-1142	1028
UCIAMS115839	50-50.5	Bulk Sediment	1765±20	1609-1822	1700
UCIAMS115840	75.0-76.0	Bulk Sediment	2680±20	2524-2860	2782
BETA179735	95.0-97.0	Bulk Sediment	2830±40	2909-3200	3043
BETA179736	146.5-147	Bulk Sediment	3710±40	3911-4234	4065
BETA179737	182.0-183.0	Bulk Sediment	4220±40	4640-5054	4815
WSU GeoAnalytical	247.0-248.5	Tsoyawata Tephra	7015±45	7521-8016	7815[1]
NSRL12147	255.0-257.0	Bulk Sediment	8720±50	8737-9759	9220

[1] Based on previously determined age of Tsoyawata Tephra[6]

Bacon 2.2, Bayesian age-depth modeling software[7] was used to create age-depth models, utilizing INTCAL13[8]. The following prior values to begin the Markov-chain Monte Carlo iterations [accumulation shape = 1.5, accumulation mean = 60 years/cm, memory strength = 4, memory mean = 0.5]. We used accumulation rate and memory mean priors that were lower than the software's pre-set values to accommodate low accumulation rate and low data resolution, as well as increase model flexibility, near the bottom of the core (Supplementary Fig. S3).

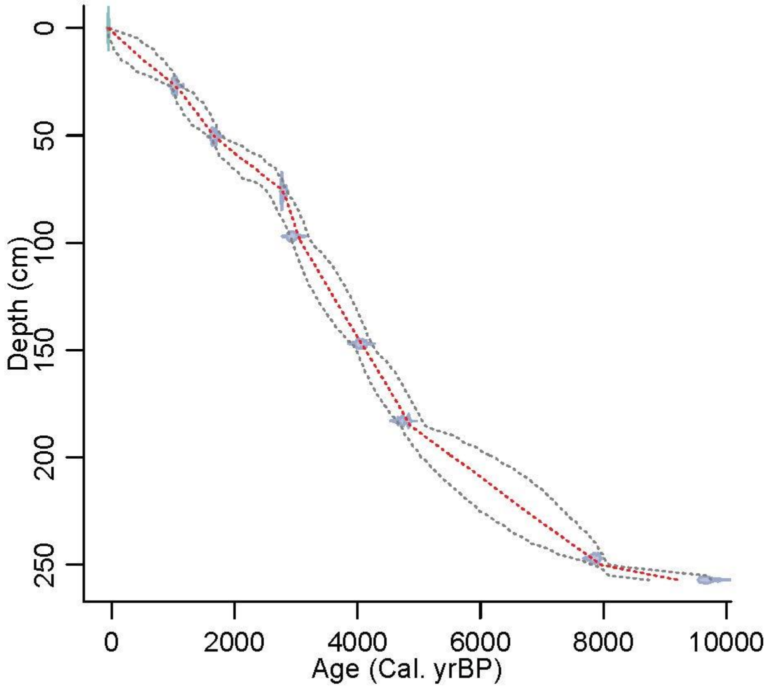

Supplementary Figure S3. Age-depth model for Kirman Lake based on Bayesian modeling of data from Supplementary Table S1. Light blue bands indicate probability distributions for calibrated ^{14}C dates. Red curve shows single 'best' model based on the weighted mean age for each depth, while grey dotted lines indicate 95% confidence interval.

Sedimentological, Geochemical and Isotopic Analysis

Loss-on-ignition (LOI) at 550°C was conducted at 1-cm intervals through the core and on Ekman-sampled surface sediments using standard procedures[9]. Stable isotope ratios and elemental concentrations of carbon and nitrogen for 20 bulk sediment samples were processed at the University of Waterloo Environmental Isotope Laboratory following standard methodology[10]. Sediment samples were rinsed with 10% hydrochloric acid to remove carbonates, and then rinsed repeatedly with de-ionized water until a neutral pH was reached.

Acid-washed samples were freeze-dried to remove moisture, and the fine fraction (<500 μm) was analysed for organic carbon and nitrogen elemental and isotope composition using a continuous flow isotope ratio mass spectrometer (CF-IRMS). The stable carbon isotope ratio of the organic fraction is reported as $\delta^{13}C$ values in per mil (‰) units relative to the Vienna-Peedee Belemnite (VPDB) standard and the nitrogen isotope ratio is expressed as $\delta^{15}N$ (‰) relative to atmospheric nitrogen standard. The analytical uncertainty for elemental carbon and nitrogen was ±0.04% and ±0.005%, respectively, while $\delta^{13}C$ and $\delta^{15}N$ values have an analytical uncertainty of ±0.04‰ and ±0.05‰, respectively. Carbon-to-nitrogen (C:N) ratios were calculated using percent dry weight organic carbon and nitrogen contents.

Diatom, Palynological, Charcoal and Malacological Analysis

Diatom samples were processed following standard procedures to isolate diatom valves for identification and enumeration[11]. Approximately 0.5 g of sediment was mixed with 10% HCl to remove any $CaCO_3$ present. Following washing with deionized water, samples were treated with concentrated nitric and sulfuric acids (50:50 ratio by molecular weight) and heated to digest organic matter. Samples were subsequently washed until neutralized. Subsamples of the diatom slurries were evaporated onto glass coverslips, and the coverslips were mounted onto glass microscope slides using Naphrax® (refractive index = 1.74). A minimum of 600 diatom valves were identified and enumerated using a Nikon Eclipse E6000 microscope equipped with Nomarski differential interference contrast (DIC) optics and a 100X oil immersion objective (total magnification = 1,000X). Diatom identifications were based on several references[12, 13, 14, 15, 16]. A summary diatom stratigraphy shows that planktonic diatoms generally increase with diatom-inferred lake depth, which is expected because increased lake level in a simple basin,

such as Kirman Lake, results in relatively more open water habitat[17] (Supplementary Fig. S4). The extended dry period during the mid-Holocene is dominated by *Gomphonema angustatum* (60%). Today in Kirman Lake *Gomphonema angustaum* lives attached to *Scirpus acutus* var. *occidentalis* (common tule), so high abundances of *Gomphonema angustaum* likely indicate greater amounts of *Scirpus*. A previously published 57-lake, Sierra Nevada calibration set was used to develop diatom-inference (D-I) models[18] to quantitatively determine lake depth and salinity, and therefore hydrological variations, from the Kirman Lake fossil diatom assemblages. Because Kirman Lake is topographically closed, decreases in effective moisture will result in lower lake levels and increased salinity[19]. The D-I models for depth and salinity were based on weighted averaging with inverse deshrinking (49 lakes and 309 taxa were included) and weighted averaging partial least squares with two components (55 lakes and 314 taxa included), respectively. For the D-I lake depth model ($r^2 = 0.84$; RMSE = 2.7 m; $RMSEP_{boot}$ = 5.6 m; $RMSEP_{jack}$ = 5.1 m), the RMSE and RMSEP values are large; however, the RMSEP as a percent of the gradient is still low (14.7 and 13.4 for $RMSEP_{boot}$ and $RMSEP_{jack}$, respectively). Based on these errors, the inferred lake depth values should be viewed with caution, although the general trends in lake depth recorded here are considered accurate[20, 21]. The D-I model for salinity presented here ($r^2 = 0.96$; RMSE = 4.23 mgL^{-1}; $RMSEP_{boot}$ = 16.34 mgL^{-1}; $RMSEP_{jack}$ = 15.00 mgL^{-1}) has a slightly larger predicted error than previous models (n = 94 diatom taxa; $r^2 = 0.96$; RMSE = 4.06; $RMSEP_{jack}$ = 11.13 mgL^{-1})[18]; however, species overlap between the modern Sierra Nevada calibration set diatoms and Kirman Lake fossil diatom assemblages is considerably greater with the new model, which includes 314 diatom taxa compared to the previous model which included 94 taxa[18]. Therefore, the reliability of D-I salinity values are substantially higher, making the model much more useful for reconstruction based on the fossil

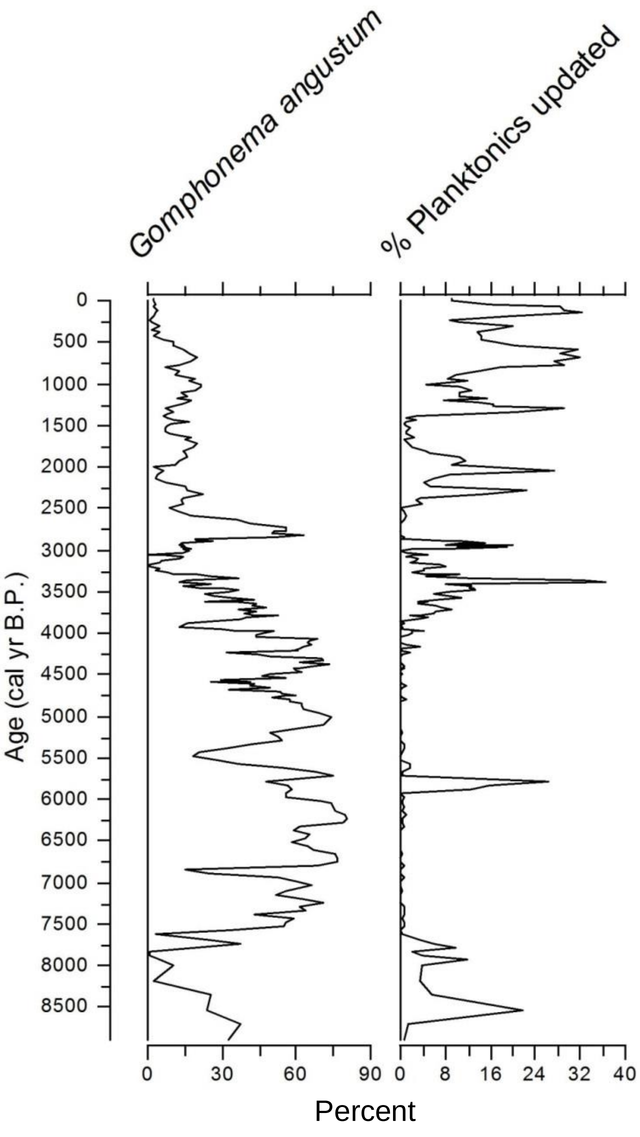

Supplementary Figure. S4. Summary diatom stratigraphy showing percentage abundances of *Gomphonema angustatum*, associated with *Scirpus*, and % Planktonics, associated with open water conditions at Kirman Lake.

diatom data from Kirman Lake. Diatom content prior to 9 ka was too low for reliable use in inference modelling.

Reliability assessment of the D-I model output values follows the numerical classification scheme in which D-I values generated with 95-100% analog between fossil and modern calibration set diatom species are likely to be very reliable, 90-95% are reliable, 75-90% may be reliable, and <75% are likely to be unreliable[22]. There was sufficient overlap between the Kirman Lake fossil diatom taxa and the Sierra Nevada calibration set diatom taxa incorporated into the D-I models to allow for reliable results, with the exception of one sample, 172.0 cm (overlap = 72.0%), which was removed from further analyses. Overlap between Kirman Lake fossil diatom taxa and the diatom taxa incorporated into the D-I models ranged from 77.0-100% (average = 97.3%) with the 255 samples classified as follows: seven samples = 75-90% (may be reliable), 20 samples = 90-95% (reliable), and 228 samples = ≥95% (very reliable)[21]. There was no clustering of the samples that were classified under each category and they were distributed throughout the core. The diatom-inference model was applied to fossil diatom assemblages identified in Kirman Lake, and inferred lake depth and salinity are shown in Fig. 2.

Pollen was concentrated from 1 ml sediment sub-samples, identified and counted using standard methods[23] and the UCLA California reference collection for assistance in identifications. A minimum of 300 identified terrestrial pollen grains were counted per-sample. Macroscopic charcoal grains were analyzed from 1 ml sediment subsamples taken at contiguous 1 cm intervals along the core using standard methods[24].

Freshwater molluscs were picked from the sediment core at 0.5 cm intervals, and identified to species using a low-power stereo microscope. Sediment was dried, soaked in sodium hexametaphosphate and washed through nested sieves where necessary. *Pisidium nitidum* and *Valvata humeralis* dominated the mollusc assemblage and were the focus of the surface sediment survey and Holocene ecological interpretation. Most *P. nitidum* shells were whole, suggesting in situ deposition. Only whole shells and intact *P. nitidum* half shells were enumerated to avoid misidentification of fragments, with counts expressed as number of individuals per cc. Ekman-sampled surface sediments were processed similarly and modern habitat preferences and vegetation associations were noted.

Data Archive

https://dataverse.harvard.edu/dataverse/UCLAGMacDonald

References

1. Smith, D. M., Scaife, A. A. & Kirtman, B. P. What is the current state of scientific knowledge with regard to seasonal and decadal forecasting. *Environ. Res. Lett.* **7**, 015602 (2012).

2. Newman, M., Compo, G.P., Alexander, M. A. ENSO-Forced Variability of the Pacific Decadal Oscillation. *J. Clim.* **16**, 3853–3857 (2003).

3. Wright Jr., H. E. Coring tips. *J. Paleolimnol*. **6**, 37-49 (1991).

4. Hunter, R.D. Effects of low pH and low calcium concentration on the pulmonate snail *Planorbella trivolis*: a laboratory study. *Can. J. Zool.* **68**, 1578-1583 (1990).

5. Brett C. E. & Baird, G. C. Comparative taphonomy: a key to paleoenvironmental interpretation based on fossil preservation. *Palaios* **22**, 205-221 (1986).

6. Bacon, C.R. Eruptive history of Mount Mazama and Crater Lake Caldera, Cascade Range, USA. *J. Volcanol. Geoth. Res.* **18**, 57-115 (1983).

7. Blaauw, M., & Christen, J. A. Flexible paleoclimate age-depth models using an autoregressive gamma process. *Bayesian Analysis* **6**, 457-474 (2011).

8. Reimer, P. J., *et al.* IntCal13 and Marine13 radiocarbon age calibration curves 0-50,000 years cal BP. *Radiocarbon* **55**, 1869-1887 (2013).

9. Heiri, O., Lotter, A. F., & Lemcke, G. Loss on ignition as a method for estimating organic and carbonate content in sediments: reproducibility and comparability of results. *J. Paleolimnol.* **25**, 101-110 (2001).

10. Wolfe, B. B., Edwards, T. W., Elgood, R. J., & Beuning, K. R. Carbon and oxygen isotope analysis of lake sediment cellulose: Methods and applications in *Tracking environmental change using lake sediments Vol. 2 Physical and geochemical methods* (eds Last, W.M. & Smol, J.P.) 373-400 (Kluwer Academic Publishers, 2001).

11. Battarbee, R. W. *et al.* Diatoms in *Tracking environmental change using lake sediments Vol. 3: Terrestrial, algal and siliceous indicators* (eds. Smol, J.P., Birks, H.J. & Last, W.M.) 155-202 (Kluwer Academic Publishers, 2001).

12. Krammer, K., & Lange-Bertalot, H. *Bacillariophyceae: English and French translation of the keys* Vol. 5. (Spektrum Akademischer Verlag Gmbh, 2000).

13. Cumming, B. F., Wilson, S. E., & Hall, R. I. *Diatoms from British Columbia (Canada) lakes and their relationship to salinity, nutrients and other limnological variables*. (Bibliotheca diatomologica, 1995).

14. Camburn, K. E., & Charles, D. F. *Diatoms of low-alkalinity lakes in the northeastern United States*. (Academy of Natural Sciences, 2000).

15. Fallu, M. A., Allaire, N., & Pienitz, R. *Freshwater diatoms from northern Québec and Labrador (Canada): Species-environment relationships in lakes of boreal forest, forest-tundra and tundra regions* Vol. 45. (J. Cramer, 2000).

16. Moser, K., Smol, J. P., & MacDonald, G. M. *The ecology and distribution of diatoms in Wood Buffalo National Park, Northern Alberta and the Northwest Territories, Canada*. (The Academy of the Natural Sciences of Philadelphia, 2004).

17. Wolin, J. A. & Duthie, H.C. Diatoms as indicators of water-level changes in freshwater lakes in *The diatoms: applications for the earth and environmental sciences* (eds Stoermer, E.F. & Smol, J.P.) 183-204 (Cambridge University Press, 1999).

18. Bloom, A. M., Moser, K. A., Porinchu, D. F., & MacDonald, G. M. Diatom-inference models for surface-water temperature and salinity developed from a 57-lake calibration set from the Sierra Nevada, California, USA. *J. Paleolimnol*. **29**, 235-255 (2003).

19. Fritz, S. C., Cumming, B. F., Gasse, F. & Laird, K. R. Diatoms as indicators of hydrologic and climatic change in saline lakes in (eds Stoermer, E.F. & Smol, J.P.) *The diatoms: applications for the environmental and earth sciences* 41-72 (Cambridge University Press, 1999).

20. Birks, H. J. B. (1998). DG Frey and ES Deevey Review 1: Numerical tools in palaeolimnology-progress, potentialities, and problems. *J. Paleolimnol*. **20**, 307-332

21. Moser, K. A. *et al*. Paleohydrology inferred from diatoms in northern latitude regions. *J. Paleolimnol.* **24**, 93-107 (2000).

22. Bradshaw, E. G., Jones, V. J., Birks, H. J. B., & Birks, H. H. Diatom responses to late-glacial and early-Holocene environmental changes at Kråkenes, western Norway. *J. Paleolimnol*. **23**, 21-34 (2000).

23. Faegri, K., Kaland, P. E., & Krzywinski, K. *Textbook of pollen analysis* . (John Wiley & Sons Ltd., 1989).

24. Whitlock, C., & Larsen, C. Charcoal as a fire proxy. In Tracking environmental change using lake sediments (75-97). (Springer Netherlands, 2001)..
